# Supplementary material for: Progesterone Receptor Modulates Extraembryonic Mesoderm and Cardiac Progenitor Specification during Mouse Gastrulation
Source: Int J Mol Sci. 2022 Sep 7;23(18):10307. doi: 10.3390/ijms231810307 (PMC9499561; doi:10.3390/ijms231810307)
Supplement: Supplementary file 1 [file ijms-23-10307-s001.zip › ijms-1882061-supplementary.pdf]

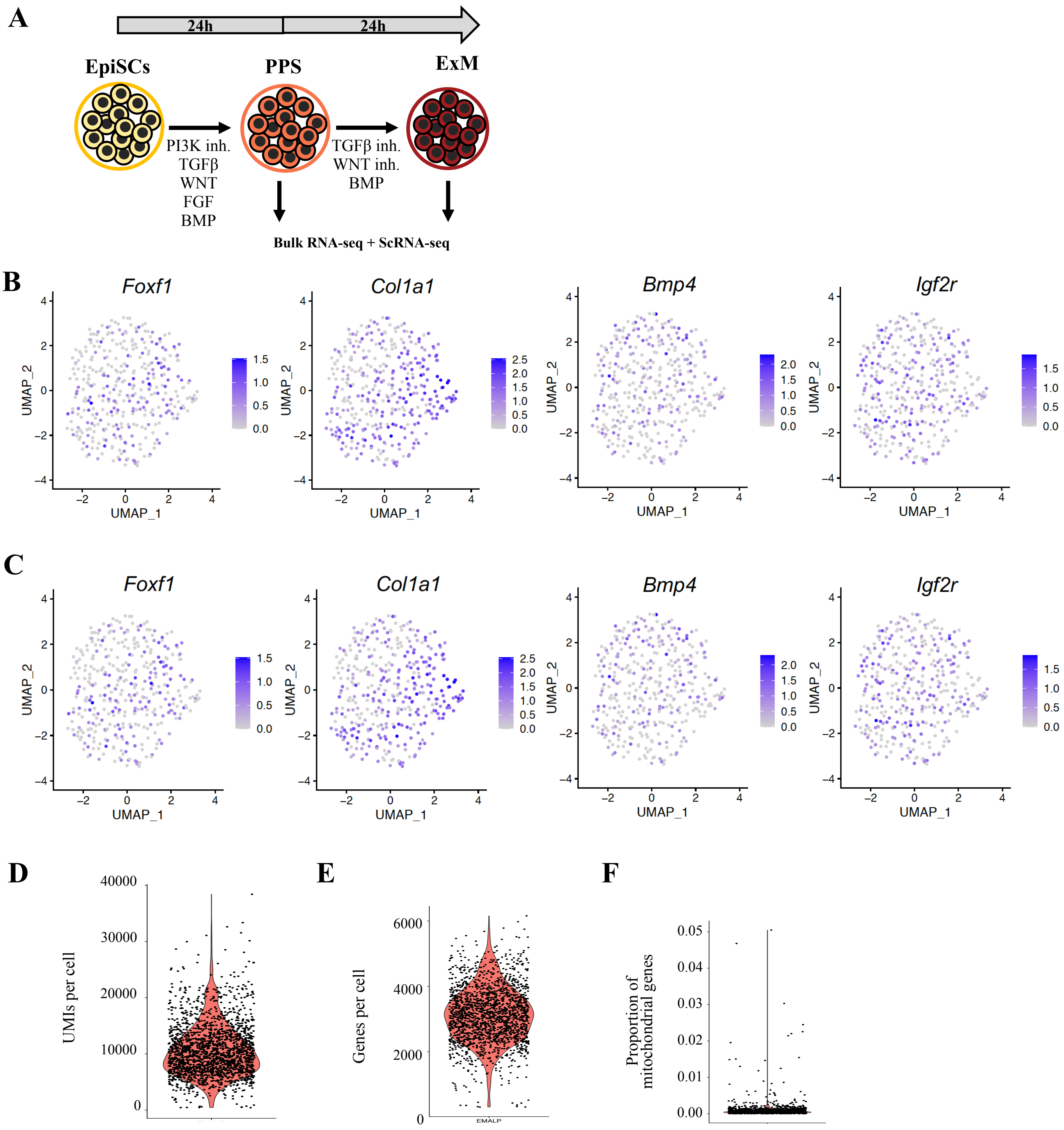

Supplementary Figure S1. High levels of BMP restrict the fate of PS cells towards ExM. (**A**) Illustration summarizing the protocol used to differentiate EpiSCs towards PPS and ExM cells. **(B)** UMAP plots of PPS. Individual cells are coloured by expression of key posterior primitive streak signature genes. (**C**) UMAP plots of ExM. Individual cells are coloured by expression of key extraembryonic mesoderm markers. (**D**) Violin plot reporting the number of UMI per cell. (**E**) Violin plot illustrating the distribution of genes per cell. (**F**) Violin plot reporting the percentage of mitochondrial genes in the scRNA-Seq dataset.

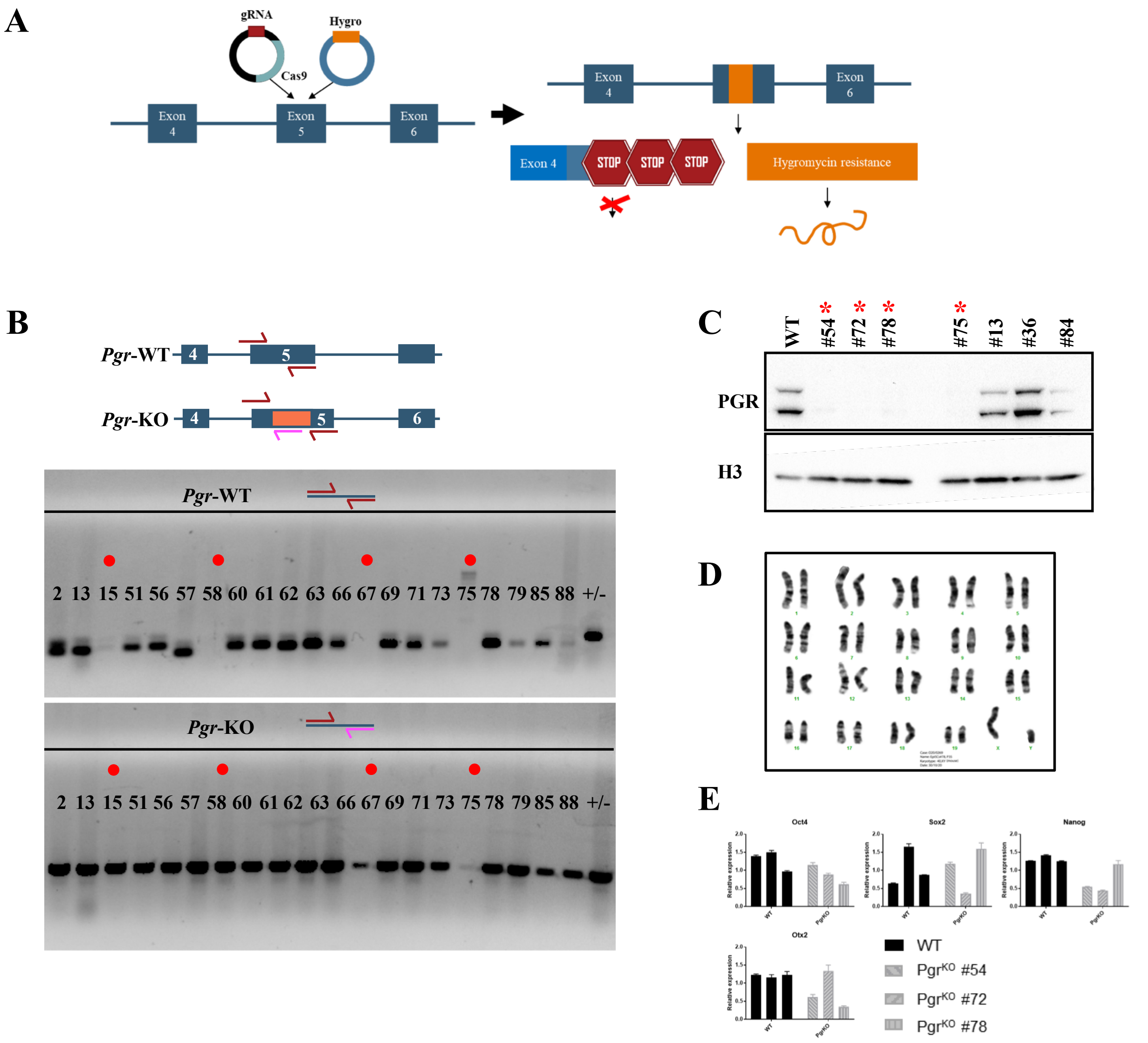

Supplementary Figure S2. Derivation and characterization of *Pgr-KO* EpiSC clones. (**A**) Schematic illustration of the strategy to generate the targeting construct to produce *Pgr-KO* EpiSCs. The exon 5 of *Pgr* gene was targeted using CRISPR/Cas9 and a hygromycin resistance cassette, that includes promoter and poly(A) signal. The targeting construct was knocked-in in exon 5 of *Pgr* disrupting the production of the protein. Low passage EpiSCs were transfected with gRNA and Cas9 constructs. After hygromycin selection, the clones are genotyped. (**B**) Upper panel: Illustration of the PCR genotyping strategy with primers specific to either WT (brown) or targeting construct (left KO, pink). Lower panel: PCR results showing the candidate *Pgr*$^{-/-}$ (KO) clones. The absence of WT band indicates inactivation of both *Pgr* alleles (red circle). (**C**) Western blot analysis confirmed absence of PGR protein in *Pgr*$^{-/-}$ (KO) among selected clones (indicated by red asterisks) generated by CRISPR/Cas9 approach in EpiSCs. Some of the lines are heterozygous knock-in, with one allele containing the hygromycin cassette and the second allele being targeted by the CAS9 protein (data not shown). Lines #54, #72, and #78 were chosen for the follow-up experiments. (**D**) Line #78 chosen for karyotype analysis, showed correct G-band distribution and chromosome numbers. (**E**) Relative mRNA expression of pluripotency (*Oct4*, *Sox2*, and *Nanog*) and EpiSCs (*Otx2*, *Fgf5*) signature markers measured by RT-qPCR on selected clones, confirming the pluripotency after clone re-derivation.

**Criteria**	**Genes that pass the criteria**
TFs enriched in EpiSCs compared to mESCs with our culture conditions (Figure 1)	*Npas1*, *Ikzf4*, *Prox1*, *Lats2*, *Nkx3-1*, *Zfp521*, *Zfhx4*, *Pgr*, *Meis3*, *Tshz1*, *Bcl11b*, *Bmyc*, *Zfp579*, *Prdm9*, *Creb5*, *Zfp575*, *Foxe1*, *Zhx2*, *Zxdb*
TFs enriched in EpiSCs compared to mESCs in publish data sets (Sugimoto et al. Stem Cell Reports 2015 [67])	*Npas1*, *Prox1*, *Lats2*, *Zfp521*, *Zfhx4*, *Pgr*, *Meis3*, *Tshz1*, *Bcl11b*, *Bmyc*, *Zfp579*, *Prdm9*, *Zxdb*
KO mouse phenotype that includes embryonic death and abnormalities affecting mesoderm derived tissues/cells (ref).	*Prox1*, *Zfp521*, *Zfhx4*, *Pgr*,*Tshz1*, *Bcl11b*

Supplementary Table S1. Criteria for shortlisting genes that could prime mesoderm differentiation.

Target	Forward primer (5’->3’)	Reverse primer (5’->3’)
Pgr LBD	CTATGCAGGGCATGACAACA	TTGGTTGAGACTGGTCAGCA
PgrB	GGAGCTTTCTCTGGGGTAGAAG	CGGGGCTCTGGAATTTCT
Pgr 3’LBD	CCTGGCTTGAAGATCAAGGA	GGAAGTCTGGGAAGGTGGA
Pgr 3’UTR	TGACATTATTGAGGGCAGGTT	CACAATTTCTGTCTATTCACAGGTCT
Hand1	CAAGCGGAAAAGGGAGTTG	GTGCGCCCTTTAATCCTCTT
Flk1	CAGTGGTACTGGCAGCTAGAAG	ACAAGCATACGGGCTTGTTT
Foxf1	AGCATCTCCACGCACTCC	TGTGAGTGATACCGAGGGATG
Hand2	CCGACACCAAACTCTCCAA	GATCCATGAGGTAGGCGATG
Nkx2.5	CGCCTTTCTCAGTCAAAGACA	CAGACAGGTCCCCAGACG
Gata4	TTCGCTGTTTCTCCCTCAAG	CAATGTTAACGGGTTGTGGA
Tnni1	GAATGTGGAGGCTATGTCTGG	TGTCATACAGCAAGCCAACC
Myl7	CAGGGGGTGGTGAACAAG	GTGTCAGCGCAAACAGTTG
Tbx3	TTGCAAAGGGTTTTCGAGAC	TGCAGTGTGAGCTGCTTTCT
Pou5f1	GTTGGAGAAGGTGGAACCAA	CTCCTTCTGCAGGGCTTTC
Sox2	GGCAGAGAAGAGAGTGTTTGC	TCTTCTTTCTCCCAGCCCTA
Nanog	GGACAGGTTTCAGAAGCAGAA	GGTTTTGAAACCAGGTCTTAACC
Otx2	GGTATGGACTTGCTGCATCC	CTCTCCCTTCGCTGTTTCC
Fgf5	ACCGGTGAAACCAAAGGTG	GCGAAACTTCAGTCTGTACTTCACT
Fn1	GGAATGGACCTGCAAACCTA	GTAGGGCTTTTCCCAGGTCT
Tbp	GGGGAGCTGTGATGTGAAGT	CCAGGAAATAATTCTGGCTCA
Sdha	TGTTCAGTTCCACCCCACA	TCTCCACGACACCCTTCTG

Supplementary Table S2. Primers used for RT-qPCR.
